# Xenon isotopes reveal a geomagnetic prelude to Earth’s oxygenation

**DOI:** 10.1093/nsr/nwag172

**Published:** 2026-03-17

**Authors:** Yong Wei

**Affiliations:** Institute of Geology and Geophysics, Chinese Academy of Sciences, China; School of Space Exploration, University of Chinese Academy of Sciences, China

## Abstract

Ancient xenon isotopes reveal that a temporary weakening of Earth’s magnetic field enhanced hydrogen escape to prime the atmosphere for oxygenation, before its recovery locked in the Great Oxidation Event. More than simple shields, magnetic fields may act as an active filter for atmospheric evolution, with consequences for planetary habitability and the divergent histories of Earth and Mars.

The Great Oxidation Event (GOE; ~2.45–2.30 Ga) marks the first sustained rise of atmospheric O₂ and a fundamental reorganization of Earth’s surface redox cycling. Although oxygenic photosynthesis may have originated hundreds of millions of years earlier, the timing of the GOE remains debated: why did atmospheric oxygen accumulate when it did, and not before? Proposed explanations range from evolving volcanic and metamorphic reductant fluxes to nonlinear photochemical feedbacks, yet most treat atmospheric escape—the irreversible loss of hydrogen to space, which net-oxidizes the surface—as a smoothly varying background parameter tied to solar forcing or atmospheric composition [1–3]. If, however, escape efficiency changed episodically rather than monotonically, it could modulate the redox balance without requiring equally abrupt changes in surface fluxes. Escape is ultimately governed by the magnetic shield that stands between a planet’s atmosphere and the solar wind—a shield whose strength depends on processes originating deep in the planetary interior. Here, using the isotopic and elemental record of atmospheric xenon, I show that a transient enhancement in atmospheric escape efficiency centered at ~2.62 Ga—directly preceding the GOE—is the most parsimonious explanation of the combined noble-gas record, and I discuss the geophysical processes that may have opened and closed this window.

Xenon is uniquely diagnostic among noble gases because its atmospheric inventory bears the imprint of two distinct escape signatures. First, modern atmospheric Xe is isotopically heavy relative to plausible primordial compositions, implying prolonged mass-dependent fractionation (MDF, that is, preferential loss of lighter isotopes). Archean and Paleoproterozoic fluid inclusions and sedimentary rocks preserve intermediate MDF values, documenting a gradual evolution toward the modern isotopic composition that appears to terminate after ~2.0 Ga [4–6]. Second, atmospheric Xe is strongly depleted relative to Kr compared with chondritic or solar compositions, a selective depletion that neutral hydrodynamic escape alone struggles to explain but that ion escape pathways can accommodate because Xe is readily ionized and couples to magnetospheric plasma outflows [7]. These two records—Xe MDF as a function of time and the Xe/Kr ratio—are sensitive to different moments of the escape-efficiency history and, when inverted jointly, can constrain not only the long-term average but also the temporal structure of escape.

I performed a joint inversion of 16 published Xe MDF measurements spanning 3.5–0.3 Ga (screened for age reliability and analytical quality; see Supplementary Information for screening criteria, age determinations, and uncertainty treatment) and three independent Xe/Kr constraints, testing models with zero, one, or two transient escape-efficiency enhancements (‘windows’) superimposed on pre- and post-GOE background states. Each model maps a parameterized escape-efficiency history into predicted Xe MDF evolution and Xe/Kr evolution via shared physical parameters, with intrinsic scatter terms for each dataset. Information-criterion model selection (Akaike information criterion, AIC; Bayesian information criterion, BIC) decisively favors the single-window model (*N* = 1; AIC = 86.05) over the background-only model (*N* = 0; AIC = 90.71), while the two-window model (*N* = 2) is disfavored by BIC (102.98 vs. 95.49), indicating that a second window is not justified at current data density. The preferred window is centered at 2.62 ± 0.05 Ga, with a duration of ~161 Myr and moderate amplitude. Bootstrap resampling (10 000 iterations) confirms that the center time is the most robust parameter; amplitude and duration trade off against each other, as expected for an integrated constraint: the Xe record captures the cumulative effect of enhanced escape over a finite interval rather than pinpointing an instantaneous event. Importantly, I also tested whether a simple linear decline in Xe MDF from 3.5 to 2.0 Ga—without any discrete window—could explain the data; this monotonic model is rejected by the information criteria because it cannot simultaneously satisfy the Xe/Kr constraint, which requires a period of enhanced *selective* (ion) escape rather than a gradual uniform trend. Error bars in Fig. 1 represent 1σ analytical uncertainties on both MDF values and ages.

**Figure 1.**
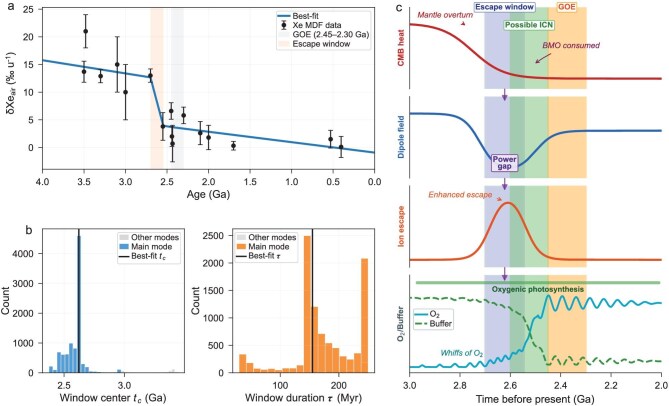
Xenon isotope constraints and geophysical context for a transient escape-efficiency window. (a) Xenon mass-dependent fractionation (MDF, expressed as per-amu deviation from modern atmospheric Xe) versus time for 16 Archean–Proterozoic samples (filled circles with 1σ error bars on both fractionation and age). The solid curve shows the best-fit single-window model (*N* = 1). A vertically shaded band marks the inferred escape window (center 2.62 ± 0.05 Ga, duration ~161 Myr); another shaded band marks the GOE interval (2.45–2.30 Ga). (b) Bootstrap distributions (10 000 iterations) of the escape-window center time *t*_c_ (left) and duration τ (right). The principal mode is distinguished from secondary modes by shading; vertical black lines mark the best-fit values. The center time is the most robust parameter; amplitude and duration trade off against each other, as expected for an integrated constraint. (c) Schematic causal chain linking CMB processes to atmospheric oxygenation over the 3.0–2.0 Ga interval. From top to bottom: CMB heat flow, showing the decline following mantle overturn and consumption of the basal magma ocean; geomagnetic dipole field strength, with the inferred “power gap” between the waning thermal dynamo and the onset of compositional convection driven by inner-core nucleation (ICN); ion escape efficiency, peaking during the field minimum; and atmospheric O_2_ with the reductant buffer shown as a dashed curve, showing pre-GOE “whiffs” of oxygen followed by irreversible oxygenation. Vertical bands mark the escape window, ICN interval, and GOE. Arrows indicate the direction of causal influence between lanes.

What physical mechanism could open—and subsequently close—such a window at ~2.62 Ga? The escape efficiency of ionized species from Earth’s upper atmosphere is governed largely by the geometry and strength of the geomagnetic field, which controls polar-cap area, magnetospheric convection, and the fraction of solar-wind energy coupled into the ionosphere. Comparative observations of Earth and Mars during the same corotating interaction region passage demonstrate that, under a comparable increase in solar-wind dynamic pressure, the rate of increase in Martian O⁺ outflow flux was one order of magnitude higher than that on Earth, implying that a strong planetary dipole effectively prevents coupling of solar-wind kinetic energy to planetary ions [8]. By extension, a substantially weakened or more multipolar paleomagnetic field in Earth’s past would have reduced this shielding effect, allowing far greater ion escape on geological timescales.

Recent geodynamo modeling provides a compelling candidate for such a field weakening. Wang and Wu [9] show that water-induced mantle overturn during the Archean drove anomalously high heat flow across the core–mantle boundary (CMB), powering a vigorous thermally driven dynamo that explains the elevated paleointensity records from ~3.5 to 2.5 Ga [10,11]. Crucially, their model predicts that as the basal magma ocean was progressively consumed, CMB heat flow declined from ~2.7 Ga onward, weakening the thermal dynamo. This predicted decline aligns with the opening of the xenon escape window. Moreover, the intense core cooling driven by the overturn may have accelerated the approach to inner-core nucleation conditions: if the overturn simultaneously exhausted its own fuel and pushed the core toward the liquidus, then the decline of the thermal dynamo and the onset of compositional convection driven by inner-core crystallization are not independent coincidences but causally linked stages of a single process. The escape window would then record the transient interval—a geodynamo ‘power gap’—between the death of one dynamo driver and the birth of another. I emphasize that inner-core nucleation timing remains debated, with estimates ranging from the Neoarchean to the Neoproterozoic [10,12]; the xenon constraint offered here is independent of paleomagnetic intensity data and provides a testable atmospheric boundary condition for geodynamo models.

The geochemical consequence of this escape window is direct. Enhanced hydrogen escape during the window irreversibly removed reducing power from the surface–atmosphere system, progressively eroding the reductant buffer (reduced volcanic gases, dissolved Fe²⁺) that had suppressed O₂ accumulation throughout the Archean. The ~200 Myr lag between the escape-window centroid (~2.62 Ga) and the canonical GOE onset (~2.45 Ga) is consistent with the timescale required to titrate this buffer to exhaustion. During the late stages of buffer depletion, transient ‘whiffs’ of oxygen become possible—local or short-lived oxidation events that appear in the proxy record before global stabilization [13]—naturally explained as the system flickering near a tipping point. Once the buffer is consumed, the methane greenhouse collapses, hydrogen escape is throttled by the rise of O₂ itself (the ‘oxygen valve’ [14]), and the atmosphere locks into an oxygenated state. The escape window thus does not cause the GOE directly; rather, it preconditions the atmosphere by irreversibly depleting the reductant reservoir, setting the stage for the nonlinear transition.

Three testable predictions follow. First, high-quality paleointensity data in the 2.8–2.5 Ga interval should reveal a decline in virtual dipole moment and possibly increased reversal frequency, consistent with the dynamo power gap. Second, additional Xe MDF and Xe/Kr measurements in the 2.8–2.4 Ga interval—from fluid inclusions in well-dated hydrothermal quartz or barite—would sharpen the shape and duration of the window. Third, coupled geodynamo–magnetosphere–escape simulations, driven by the CMB heat-flow history of Wang and Wu [9], should be able to reproduce the inferred escape enhancement quantitatively. A planetary comparison brings the argument full circle. I noted at the outset that the magnetic shield linking a planet’s deep interior to its atmospheric fate is the critical variable; Mars now provides a decisive test case. InSight seismic data reveal that Mars possesses a solid inner core with a radius of ~613 km [15], demonstrating that core crystallization is not unique to Earth. Yet, the Martian dynamo still ceased early in the planet’s history, and without a sustained magnetic shield, Mars suffered permanent atmospheric stripping—with ion escape rates far exceeding those of a dipole-shielded planet under equivalent solar-wind forcing [8]. The Martian inner core tells us that crystallization alone does not guarantee dynamo survival; what matters is whether compositional convection ignites before the preceding thermal dynamo expires. On Earth, mantle-overturn-driven cooling may have created precisely this condition—pushing the core toward nucleation while the thermal dynamo still flickered—so that the power gap remained a transient window rather than a permanent collapse. The xenon escape window is the atmospheric scar of that narrow passage. Had the transition failed, as it did on Mars, Earth’s atmosphere might have been stripped beyond recovery, and the GOE would never have occurred. The oxygenation of our planet, it appears, was as much a geophysical event as a biological one—its fate decided not at the surface, but at the boundary between core and mantle, thousands of kilometers below.

## Supplementary Material

nwag172_Supplemental_File
