# Supplementary material for: Xenon isotopes reveal a geomagnetic prelude to Earth’s oxygenation
Source: Natl Sci Rev. 2026 Mar 17;13(7):nwag172. doi: 10.1093/nsr/nwag172 (PMC13105161; doi:10.1093/nsr/nwag172)
Supplement: nwag172_Supplemental_File [file nwag172_supplemental_file.pdf]

Supplementary Information

Xenon isotopes reveal a geomagnetic prelude to Earth's oxygenation

Yong Wei[1,2]*

1 Institute of Geology and Geophysics, Chinese Academy of Sciences, Beijing, China

2 School of Space Exploration, University of Chinese Academy of Sciences, Beijing, China

Email: [weiy@mail.iggcas.ac.cn]

S1. Data Compilation and Screening Criteria

S1.1 Xenon mass-dependent fractionation (MDF) data

The joint inversion uses 16 published Xe MDF measurements spanning 3.5–0.3 Ga, drawn from Archean and Paleoproterozoic fluid inclusions and sedimentary rocks. Here, the compiled Xe MDF values are treated consistently as Xe isotopic fractionation relative to modern atmospheric Xe, expressed in ‰ per amu. The full compilation, including rejected samples, is provided in Table S1. Screening criteria are as follows:

1. **Age reliability.** Only samples with well-constrained depositional or trapping ages were retained. Acceptable age constraints include U-Pb zircon ages of interbedded volcanic units, well-established stratigraphic correlations, or direct dating of hydrothermal events (e.g., Rb-Sr or Ar-Ar ages of associated minerals). Samples with ambiguous or poorly constrained ages (e.g., the Timmins sample, which lacks a reliable independent age) were excluded.

2. **Analytical quality.** Samples with 1σ analytical uncertainties on Xe isotopic fractionation exceeding 6‰ per amu were excluded, as were measurements where mass-interference corrections or blank contributions dominated the signal.

3. **Host-phase integrity.** Preference was given to measurements from well-characterized host phases—fluid inclusions in hydrothermal quartz or barite—over bulk-rock analyses susceptible to post-depositional alteration or atmospheric contamination. Where multiple measurements existed for the same locality and age, the most recent and analytically robust determination was used.

4. **Age cutoff.** Samples younger than 0.4 Ga were excluded from the primary inversion because the Xe MDF evolution is essentially complete by ~2.0 Ga and younger samples provide no additional constraint on the escape-efficiency history. The Rhynie chert (0.404 Ga) is retained as a near-modern anchor.

S1.2 Xe/Kr data

Three independent Xe/Kr constraints are used:

- **Barberton (3.3 Ga):** $^{130}\mathrm{Xe}/^{84}\mathrm{Kr} = 0.0120 \pm 0.0020$, from fluid inclusions in hydrothermal barite [6].

- **Fortescue (2.7 Ga):** $^{130}\mathrm{Xe}/^{84}\mathrm{Kr} = 0.0126 \pm 0.0022$, from fluid inclusions in hydrothermal quartz [6].

- **Modern atmosphere (0.0 Ga):** $^{130}\mathrm{Xe}/^{84}\mathrm{Kr} = 0.0050 \pm 0.0005$, from the well-determined present-day atmospheric composition.

The Xe/Kr ratio is expressed in logarithmic form for the inversion.

S1.3 Data tables

Table S1. Xe MDF compilation (screened, 16 samples used in inversion) [4].

Sample	Age (Ga)	Age uncertainty (Ga)	Xe fractionation relative to modern air (‰ amu^{-1})	Uncertainty (‰ amu^{-1})	Age method
North Pole	3.48	0.09	21.0	3.0	U-Pb zircon (interbedded volcanics)
North Pole	3.50	0.08	13.7	1.9	U-Pb zircon (interbedded volcanics)
North Pole	3.10	0.40	15.0	5.0	Stratigraphic correlation
North Pole	3.00	0.20	10.0	5.0	Stratigraphic correlation
Barberton	3.30	0.05	12.9	1.2	U-Pb zircon
Fortescue	2.70	0.08	13.0	1.2	U-Pb zircon
Quetico Belt	2.55	0.15	3.8	2.5	Regional geochronology
Vetreny Belt	2.45	0.08	6.6	1.5	Rb-Sr / stratigraphic
Isua	2.30	0.30	5.8	1.5	U-Pb zircon
Carnaiba	2.00	0.10	1.8	2.2	Ar-Ar hydrothermal
Gaoua	2.10	0.07	2.6	2.1	U-Pb zircon
Seidorechka (FD1A)	2.441	0.0016	2.0	1.8	U-Pb zircon (high-precision)
Polisarka (FD3A)	2.434	0.0066	0.7	3.3	U-Pb zircon (high-precision)
Caramal	1.70	0.08	0.32	0.78	U-Pb zircon
Avranches	0.53	0.01	1.5	1.6	Biostratigraphy / U-Pb
Rhynie	0.404	0.001	0.1	1.9	Ar-Ar / U-Pb

Table S2. Xe/Kr data [6].

Locality	Time (Ga)	$^{130}\mathrm{Xe}/^{84}\mathrm{Kr}$	Uncertainty
Barberton	3.3	0.0120	0.0020
Fortescue	2.7	0.0126	0.0022
Modern atmosphere	0.0	0.0050	0.0005

S2. Model Description

S2.1 Overview

The model maps a parameterized escape-efficiency history $\varepsilon(t)$ into two observable time series:

1. **Xe MDF** (Xe isotopic fractionation relative to modern atmospheric Xe as a function of sample age), driven by cumulative mass-dependent isotopic fractionation proportional to the integrated escape flux.
2. **Xe/Kr ratio** ($\ln(^{130}\mathrm{Xe}/^{84}\mathrm{Kr})$ as a function of time), driven by cumulative selective ion loss of Xe relative to Kr.

Both predictions share the same underlying escape-efficiency function $\varepsilon(t)$, ensuring physical consistency.

S2.2 Escape-efficiency parameterization

The escape efficiency $\varepsilon(t)$ is modeled as a piecewise-constant background with optional rectangular transient windows:

$$\varepsilon(t) = \begin{cases} \varepsilon_{\mathrm{post}} & \text{if } t \le t_{\mathrm{GOE}} \\ \varepsilon_{\mathrm{pre}} & \text{if } t > t_{\mathrm{GOE}} \end{cases} + \sum_{j=1}^{N} A_j \cdot \mathbf{1}\left[t_{c,j} - \frac{\tau_j}{2} \le t \le t_{c,j} + \frac{\tau_j}{2}\right] \qquad (1)$$

where:

- $\varepsilon_{\mathrm{pre}}$ is the background escape efficiency before the GOE (i.e., for ages $t > t_{\mathrm{GOE}}$),
- $\varepsilon_{\mathrm{post}}$ is the background escape efficiency after the GOE (for ages $t \le t_{\mathrm{GOE}}$),
- $t_{\mathrm{GOE}} = \frac{1}{2}(2.45 + 2.30) = 2.375$ Ga is the midpoint of the GOE interval,
- N is the number of transient escape windows (tested for $N = 0,1,2$),
- A_j, $t_{c,j}$, τ_j are the amplitude, center time, and duration of the j-th window,
- $\mathbf{1}[\cdot]$ is the indicator function.

The convention adopted throughout is that t denotes **age before present** in Ga, so that $t = 3.5$ Ga corresponds to the oldest samples and $t = 0$ corresponds to the present day.

S2.3 Cumulative escape integral

The key physical quantity linking escape efficiency to observables is the **cumulative escape integral** evaluated at the observation time t_{obs}:

$$I(t_{\mathrm{obs}}) = \int_0^{t_{\mathrm{obs}}} \varepsilon(t)\, dt \qquad (2)$$

This integral represents the total integrated escape flux experienced by the atmosphere from the present day ($t = 0$) back to the time of sample formation ($t = t_{\mathrm{obs}}$). For the piecewise-constant background, the integral evaluates analytically:

$$I_{\mathrm{bg}}(t_{\mathrm{obs}}) = \begin{cases} \varepsilon_{\mathrm{post}} \cdot t_{\mathrm{obs}} & \text{if } t_{\mathrm{obs}} \le t_{\mathrm{GOE}} \\ \varepsilon_{\mathrm{post}} \cdot t_{\mathrm{GOE}} + \varepsilon_{\mathrm{pre}} \cdot (t_{\mathrm{obs}} - t_{\mathrm{GOE}}) & \text{if } t_{\mathrm{obs}} > t_{\mathrm{GOE}} \end{cases} \qquad (3)$$

For each rectangular window with parameters $(A_j, t_{c,j}, \tau_j)$, the contribution to the integral is:

$$I_{\mathrm{win},j}(t_{\mathrm{obs}}) = A_j \cdot \max\big(0,\ \min(t_{\mathrm{obs}},\ t_{c,j} + \tau_j/2) - \max(0,\ t_{c,j} - \tau_j/2)\big) \qquad (4)$$

The total integral is:

$$I(t_{\mathrm{obs}}) = I_{\mathrm{bg}}(t_{\mathrm{obs}}) + \sum_{j=1}^{N} I_{\mathrm{win},j}(t_{\mathrm{obs}}) \qquad (5)$$

S2.4 Xe MDF prediction

The Xe MDF value at observation time t_{obs} is modeled as a linear function of the cumulative escape integral:

$$y_{\mathrm{Xe,pred}}(t_{\mathrm{obs}}) = \delta_0 + s \cdot I(t_{\mathrm{obs}}) \qquad (6)$$

where:

- δ_0 is the modern (present-day) Xe MDF value relative to modern atmospheric Xe (a free parameter, expected to be near zero),
- s is the sensitivity coefficient relating integrated escape to isotopic fractionation (‰ amu^{-1} per Ga of integrated escape efficiency).

S2.5 Xe/Kr prediction

The logarithmic Xe/Kr ratio at observation time t_{obs} is modeled as:

$$\ln\left(\frac{^{130}\mathrm{Xe}}{^{84}\mathrm{Kr}}\right)_{\mathrm{pred}}(t_{\mathrm{obs}}) = R_0 + k \cdot I(t_{\mathrm{obs}}) \qquad (7)$$

where:

- $R_0 = \ln(0.0050) \approx -5.2983$ is the logarithm of the modern atmospheric $^{130}\mathrm{Xe}/^{84}\mathrm{Kr}$ ratio, fixed from the present-day measurement,
- k is the differential escape coefficient for Xe relative to Kr (a free parameter).

S2.6 Summary of free parameters

For a model with N transient windows, the total number of free parameters is $7 + 3N$:

Table S3. Model parameters.

Parameter	Symbol	Transform	Bounds	Description
Log pre-GOE escape efficiency	$\ln\varepsilon_{\mathrm{pre}}$	$\varepsilon_{\mathrm{pre}} = e^{\theta_0}$	$[-9.2,\ 2.3]$	Background escape rate before GOE
Log post-GOE escape efficiency	$\ln\varepsilon_{\mathrm{post}}$	$\varepsilon_{\mathrm{post}} = e^{\theta_1}$	$[-9.2,\ 2.3]$	Background escape rate after GOE
Modern Xe MDF offset	δ_0	—	$[-10,\ 10]$	Present-day Xe fractionation value
MDF sensitivity	s	—	$[0,\ 100]$	‰ amu^{-1} per Ga of integrated escape
Log Xe/Kr escape coefficient	$\ln k$	$k = e^{\theta_4}$	$[-9.2,\ 2.3]$	Differential ion escape rate
Log intrinsic Xe scatter	$\ln\sigma_{\mathrm{Xe}}$	$\sigma_{\mathrm{Xe}} = e^{\theta_5}$	$[-9.2,\ 3.0]$	Geological scatter in Xe MDF
Log intrinsic Xe/Kr scatter	$\ln\sigma_r$	$\sigma_r = e^{\theta_6}$	$[-9.2,\ 1.0]$	Geological scatter in Xe/Kr
Log window amplitude (j)	$\ln A_j$	$A_j = e^{\theta_{7+3(j-1)}}$	$[-9.2,\ \ln 10]$	Escape enhancement factor
Window center time (j)	$t_{c,j}$	—	$[t_{\mathrm{GOE}},\ 3.5]$	Center of escape window (Ga)
Log window duration (j)	$\ln\tau_j$	$\tau_j = e^{\theta_{9+3(j-1)}}$	$[\ln 0.03,\ \ln 0.25]$	Duration of window (Ga)

S3. Likelihood Function and Regularization

S3.1 Joint log-likelihood

The joint negative log-likelihood is the sum of contributions from the Xe MDF dataset and the Xe/Kr dataset:

$$\mathcal{L}_{\mathrm{neg}}(\boldsymbol{\theta}) = -\ln p(\mathrm{data} \mid \boldsymbol{\theta}) = -\ell_{\mathrm{Xe}}(\boldsymbol{\theta}) - \ell_{\mathrm{XeKr}}(\boldsymbol{\theta}) + \mathcal{R}(\boldsymbol{\theta}) \qquad (8)$$

where $\mathcal{R}(\boldsymbol{\theta})$ is a regularization term (Section S3.3).

S3.2 Individual likelihoods

Xe MDF likelihood. For n_{Xe} data points with observed values y_i^{Xe}, analytical uncertainties σ_i^{Xe}, and predicted values $\hat{y}_i^{\mathrm{Xe}} = \delta_0 + s \cdot I(t_i)$, the effective uncertainty combines analytical and intrinsic scatter:

$$\sigma_{\mathrm{eff},i}^{\mathrm{Xe}} = \sqrt{(\sigma_i^{\mathrm{Xe}})^2 + \sigma_{\mathrm{Xe}}^2} \qquad (9)$$

The log-likelihood is:

$$\ell_{\mathrm{Xe}} = \sum_{i=1}^{n_{\mathrm{Xe}}} \left[-\frac{1}{2} \left(\frac{y_i^{\mathrm{Xe}} - \hat{y}_i^{\mathrm{Xe}}}{\sigma_{\mathrm{eff},i}^{\mathrm{Xe}}} \right)^2 - \ln \sigma_{\mathrm{eff},i}^{\mathrm{Xe}} - \frac{1}{2} \ln(2\pi) \right] \qquad (10)$$

Xe/Kr likelihood. For n_{XeKr} data points with observed log-ratios $y_i^r = \ln({}^{130}\mathrm{Xe}/{}^{84}\mathrm{Kr})_i$, uncertainties σ_i^r (propagated from the ratio uncertainty as $\sigma_i^r = \Delta_i / R_i$ where Δ_i is the absolute uncertainty and R_i is the ratio), and predicted values $\hat{y}_i^r = R_0 + k \cdot I(t_i)$:

$$\sigma_{\mathrm{eff},i}^r = \sqrt{(\sigma_i^r)^2 + \sigma_r^2} \qquad (11)$$

$$\ell_{\mathrm{XeKr}} = \sum_{i=1}^{n_{\mathrm{XeKr}}} \left[-\frac{1}{2} \left(\frac{y_i^r - \hat{y}_i^r}{\sigma_{\mathrm{eff},i}^r} \right)^2 - \ln \sigma_{\mathrm{eff},i}^r - \frac{1}{2} \ln(2\pi) \right] \qquad (12)$$

S3.3 Regularization

A weak quadratic regularization is applied to prevent physically implausible parameter values:

$$\mathcal{R}(\boldsymbol{\theta}) = \frac{1}{2} \left[\left(\frac{s}{50} \right)^2 + \left(\frac{\varepsilon_{\mathrm{pre}}}{5} \right)^2 + \left(\frac{\varepsilon_{\mathrm{post}}}{5} \right)^2 + \left(\frac{k}{5} \right)^2 + \left(\frac{\sigma_{\mathrm{Xe}}}{20} \right)^2 + \left(\frac{\sigma_r}{2} \right)^2 \right] \qquad (13)$$

For models with $N > 0$ windows, an additional prior on the log-amplitude is included:

$$\mathcal{R}_{\mathrm{win}} = \sum_{j=1}^{N} \frac{1}{2} \left(\frac{\ln A_j - \mu_{\ln A}}{\sigma_{\ln A}} \right)^2 \qquad (14)$$

where $\mu_{\ln A} = 0$ and $\sigma_{\ln A} = 1$, corresponding to a log-normal prior on A_j centered at $A = 1$ with a factor-of-e spread.

The total objective function minimized by the optimizer is:

$$F(\boldsymbol{\theta}) = -\ell_{\mathrm{Xe}}(\boldsymbol{\theta}) - \ell_{\mathrm{XeKr}}(\boldsymbol{\theta}) + \mathcal{R}(\boldsymbol{\theta}) + \mathcal{R}_{\mathrm{win}}(\boldsymbol{\theta}) \qquad (15)$$

S4. Optimization and Model Selection

S4.1 Differential evolution

The objective function $F(\boldsymbol{\theta})$ is minimized using the differential evolution (DE) global optimizer [S1] as implemented in SciPy (scipy.optimize.differential_evolution). DE is a population-based stochastic optimizer well suited to multimodal, bounded optimization problems. The settings for the main fits are:

Setting	Value
Population size	20 × (number of parameters)
Maximum iterations	1,200
Convergence tolerance (atol)	10^{-3}
Convergence tolerance (tol)	10^{-3}
Polish (local refinement)	Yes (L-BFGS-B)
Random seed	42

For each model complexity ($N = 0,1,2$), the optimizer is run independently.

S4.2 Information-criterion model selection

Model complexity is assessed using the Akaike Information Criterion (AIC) and Bayesian Information Criterion (BIC):

$$\mathrm{AIC} = 2p + 2F^* \qquad (16)$$

$$\mathrm{BIC} = p\ln n + 2F^* \qquad (17)$$

where p is the number of free parameters, $n = n_{\mathrm{Xe}} + n_{\mathrm{XeKr}}$ is the total number of data points, and $F^* = F(\boldsymbol{\theta}^*)$ is the minimized objective function value.

Table S4. Model comparison results.

Model (N)	Parameters (p)	Data points (n)	F^* (neg. log-lik. + reg.)	AIC	BIC	ΔAIC	ΔBIC
$N = 0$	7	19	38.36	90.71	97.32	4.72	1.89
$N = 1$	10	19	33.03	86.05	95.49	0.00	0.00
$N = 2$	13	19	31.49	90.56	102.98	4.57	7.40

The single-window model ($N = 1$) is decisively preferred by both AIC and BIC. The two-window model ($N = 2$) achieves a marginally lower F^* but is penalized for its additional three parameters. The background-only model ($N = 0$) is disfavored because it cannot simultaneously satisfy the Xe MDF and Xe/Kr constraints without a period of enhanced selective escape.

S4.3 Rejection of the monotonic linear-decline model

A simple linear decline in Xe MDF from 3.5 to 2.0 Ga—without any discrete escape window—is a special case of the $N = 0$ model (with appropriately chosen $\varepsilon_{\mathrm{pre}}$ and $\varepsilon_{\mathrm{post}}$). This scenario is encompassed by the $N = 0$ fit and is therefore rejected by the same information-criterion comparison.

S5. Best-Fit Parameters ($N = 1$ Model)

Table S5. Best-fit parameter values for the preferred single-window model.

Parameter	Symbol	Best-fit value	Unit
Pre-GOE background escape efficiency	$\varepsilon_{\mathrm{pre}}$	0.0696	Gyr^{-1}
Post-GOE background escape efficiency	$\varepsilon_{\mathrm{post}}$	0.0547	Gyr^{-1}
Modern Xe MDF offset	δ_0	-0.920	‰ amu^{-1}
MDF sensitivity	s	34.26	‰ amu^{-1} Gyr^{-1}
Xe/Kr differential escape coefficient	k	2.132	Gyr^{-1}
Intrinsic Xe MDF scatter	σ_{Xe}	1.055	‰ amu^{-1}
Intrinsic Xe/Kr scatter	σ_r	0.00190	—
Window amplitude	A_1	1.576	Gyr^{-1}
**Window center time**	$t_{c,1}$	**2.62**	**Ga**
**Window duration**	τ_1	**~0.161**	**Gyr**

S6. Bootstrap Uncertainty Analysis

S6.1 Procedure

To assess parameter uncertainties and the robustness of the escape-window detection, a nonparametric bootstrap resampling analysis was performed with 10,000 iterations. Each iteration proceeds as follows:

1. **Resample with replacement.** Draw n_{Xe} samples (with replacement) from the Xe MDF dataset and n_{XeKr} samples (with replacement) from the Xe/Kr dataset.

2. **Re-fit.** Fit the $N = 1$ model to the resampled data using differential evolution with ultra-fast settings (population size = 6, maximum iterations = 300, no polish). The best-fit $N = 1$ solution from the main fit is used as a warm start to accelerate convergence.

3. **Record.** Store the fitted window parameters $(A_1, t_{c,1}, \tau_1)$ and convergence diagnostics.

S6.2 Main-mode filter

Bootstrap iterations are classified as "main mode" if the fitted window center time falls within ± 0.35 Ga of the best-fit value:

$$\text{main mode:} \quad |t_{c,1}^{(b)} - t_{c,1}^{*}| \leq 0.35 \text{ Ga} \qquad (18)$$

where $t_{c,1}^{*} = 2.62$ Ga is the best-fit center time and superscript (b) denotes the b-th bootstrap iteration.

S6.3 Results

Table S6. Bootstrap summary statistics (main-mode iterations only).

Statistic	Window center t_c (Ga)	Window duration τ (Myr)
Mean	2.62	~161
Median	2.62	~155
Standard deviation	0.05	variable
2.5th percentile	~2.52	~50
97.5th percentile	~2.72	~250

Key findings:

- **The center time t_c is the most robust parameter**, with a 95% confidence interval of approximately 2.62 ± 0.10 Ga (2.5th–97.5th percentile range).
- **Amplitude and duration trade off** against each other, producing a broader marginal distribution for τ than for t_c.
- The main-mode fraction is high, confirming that the escape window is a robust feature of the data rather than an artifact of a particular data configuration.

S7. Uncertainty Treatment

S7.1 Analytical uncertainties

All error bars reported in Figure 1 and used in the inversion represent **1σ analytical uncertainties**. For Xe MDF values, these are the reported measurement uncertainties on Xe isotopic fractionation relative to modern atmospheric Xe from the original publications. For ages, these are the reported geochronological uncertainties (see Table S1 for individual values and methods).

S7.2 Age uncertainties

Age uncertainties are not directly propagated as errors on the independent variable in the likelihood function. Instead, they are accounted for in two ways:

1. **Intrinsic scatter terms** (σ_{Xe} and σ_r) absorb part of the variance introduced by age uncertainty, since an age error translates into a mismatch between the predicted and observed MDF at the nominal age.
2. **Bootstrap resampling** naturally propagates age uncertainty: when a sample with a large age uncertainty is included in a bootstrap draw, the fitted parameters adjust to accommodate the potentially misplaced data point, broadening the parameter distributions.

S7.3 Missing uncertainties

For two samples in the original compilation (North Pole at 3.50 Ga and Fortescue at 2.70 Ga), age uncertainties were not reported in the original publications. These were assigned the median age uncertainty of the remaining samples. Similarly, for samples missing Xe fractionation uncertainties (North Pole at 3.50 Ga and Belorechenskoe at 0.17 Ga), the median Xe fractionation uncertainty was assigned. Belorechenskoe was subsequently excluded by the age cutoff criterion (age < 0.4 Ga).

Supplementary References

[S1] Storn R, Price K. Differential Evolution – A Simple and Efficient Heuristic for global Optimization over Continuous Spaces. *J Glob Optim* 1997;**11**:341–59. doi:10.1023/A:1008202821328

[4] Avice G, Marty B, Burgess R et al. Evolution of atmospheric xenon and other noble gases inferred from Archean to Paleoproterozoic rocks. *Geochim Cosmochim Acta* 2018;**232**:82–100. doi:10.1016/j.gca.2018.04.018

[5] Ardoin L, Broadley MW, Almayrac M et al. The end of the isotopic evolution of atmospheric xenon. *Geochem Perspect Lett* 2022;**20**:43–7. doi:10.7185/geochemlet.2207

[6] Vayrac F, Avice G, Zhang XJ et al. Records of mantle geodynamics and atmospheric escape in Archean quartz. *Sci Adv* 2025;**11**:eaea3380. doi:10.1126/sciadv.aea3380

[7] Zahnle KJ, Gacesa M, Catling DC. Strange messenger: a new history of hydrogen on Earth, as told by xenon. *Geochim Cosmochim Acta* 2019;**244**:56–85. doi:10.1016/j.gca.2018.09.017
